# Network pharmacology, computational biology integrated surface plasmon resonance technology reveals the mechanism of ellagic acid against rotavirus

**DOI:** 10.1038/s41598-024-58301-6

**Published:** 2024-03-30

**Authors:** Jiangang Zheng, Abdul Haseeb, Ziyang Wang, Hejie Wang

**Affiliations:** 1 Department of Public Health and Preventive Medicine, Changzhi Medical College, Changzhi, 046000 Shanxi People’s Republic of China; 2 College of Veterinary Medicine, Shanxi Agricultural University, Taigu, 030801 Shanxi People’s Republic of China; 3 Institute of TCM, Xinjiang Medical University, Urumqi, 830000 Xinjiang People’s Republic of China

**Keywords:** Ellagic acid, Rotavirus, Network pharmacology, Molecular docking, Molecular dynamics simulation, Saturation mutation analysis, Surface plasmon resonance, Biochemistry, Computational biology and bioinformatics, Drug discovery

## Abstract

The target and mechanism of ellagic acid (EA) against rotavirus (RV) were investigated by network pharmacology, computational biology, and surface plasmon resonance verification. The target of EA was obtained from 11 databases such as HIT and TCMSP, and RV-related targets were obtained from the Gene Cards database. The relevant targets were imported into the Venny platform to draw a Venn diagram, and their intersections were visualized. The protein–protein interaction networks (PPI) were constructed using STRING, DAVID database, and Cytoscape software, and key targets were screened. The target was enriched by Gene Ontology (GO) and KEGG pathway, and the ‘EA anti-RV target-pathway network’ was constructed. Schrodinger Maestro 13.5 software was used for molecular docking to determine the binding free energy and binding mode of ellagic acid and target protein. The Desmond program was used for molecular dynamics simulation. Saturation mutagenesis analysis was performed using Schrodinger's Maestro 13.5 software. Finally, the affinity between ellagic acid and TLR4 protein was investigated by surface plasmon resonance (SPR) experiments. The results of network pharmacological analysis showed that there were 35 intersection proteins, among which Interleukin-1β (IL-1β), Albumin (ALB), Nuclear factor kappa-B1 (NF-κB1), Toll-Like Receptor 4 (TLR4), Tumor necrosis factor alpha (TNF-α), Tumor protein p53 (TP53), Recombinant SMAD family member 3 (SAMD3), Epidermal growth factor (EGF) and Interleukin-4 (IL-4) were potential core targets of EA anti-RV. The GO analysis consists of biological processes (BP), cellular components (CC), and molecular functions (MF). The KEGG pathways with the highest gene count were mainly related to enteritis, cancer, IL-17 signaling pathway, and MAPK signaling pathway. Based on the crystal structure of key targets, the complex structure models of TP53-EA, TLR4-EA, TNF-EA, IL-1β-EA, ALB-EA, NF-κB1-EA, SAMD3-EA, EGF-EA, and IL-4-EA were constructed by molecular docking (XP mode of flexible docking). The MMGBS analysis and molecular dynamics simulation were also studied. The Δaffinity of TP53 was highest in 220 (CYS → TRP), 220 (CYS → TYR), and 220 (CYS → PHE), respectively. The Δaffinity of TLR4 was highest in 136 (THR → TYR), 136 (THR → PHE), and 136 (THR → TRP). The Δaffinity of TNF-α was highest in 150 (VAL → TRP), 18 (ALA → GLU), and 144 (PHE → GLY). SPR results showed that ellagic acid could bind TLR4 protein specifically. TP53, TLR4, and TNF-α are potential targets for EA to exert anti-RV effects, which may ultimately provide theoretical basis and clues for EA to be used as anti-RV drugs by regulating TLR4/NF-κB related pathways.

## Introduction

Rotavirus is a genus in the *Reoviridae* family and has no capsular membrane^1^. RV has a fragment genome of about 18,500 bp, containing 11 double-stranded RNA molecules encoding 6 structural proteins and 6 non-structural proteins^2^. Rotavirus strains are divided into 10 groups A–J, and Group A is the main pathogen causing acute gastroenteritis in infants and young children^3–5^. Rotavirus is one of the 13 diarrheal pathogens published in the 2016 global burden of disease study^6^.

In 2015, about 146,000 children under 5 years of age died from diarrhea, of which rotavirus infections accounted for 29.3%. In 2015, Weldegebriel et al. found that rotavirus seropositivity accounted for 25% of total diarrhea in 14 countries in East and South Africa^7^. Lestari et al. analyzed the main causes of acute gastroenteritis in infants and young children in Southeast Asian countries from 2008 to 2018, and 40.78% of the morbidity and death were caused by RV infection^8^. Cohen et al. collected 29,502 cases of diarrhea in 28 low- and middle-income countries around the world, of which 33.3% were caused by rotavirus infection^9^, posing a huge challenge to world public health^10^.

Currently, there are four RV vaccines certified by WHO, but rotavirus vaccines have not been included in immunization programs in developing countries^11^. Although the use of the vaccine has reduced the incidence of rotavirus, strains derived from the rotavirus vaccine have also begun to spread. At present, there are no specific drugs for RV infection, so the screening and development of anti-RV drugs are in urgent need^12^.

Pomegranate peel is often used in TCM to treat digestive tract diseases, which has been recorded in ancient medical books such as *Puji Prescription* and *Lei Gong's Gun Acupuncture Theory*. Pomegranate peel is rich in tannins, flavonoids, polysaccharides, alkaloids, and other active substances, among which tannins have the highest content, such as punicalagin and ellagic acid^13^. Punicalagin is a condensed form of ellagic acid that cannot be absorbed directly into the bloodstream by the intestine. It is first hydrolyzed before it can be absorbed and utilized by the human body^14^. Ellagic acid mainly has anti-inflammatory, antibacterial, antiviral, and anti-tumor pharmacological effects, and is a natural inhibitor of NF-κB^15–22^. Studies have shown that ellagic acid can relieve diarrhea, so it has a very high potential efficacy for anti-RV^23^.

With the development of bioinformation technologies such as network pharmacology, molecular docking, and molecular dynamics simulation, drug screening and development are no longer dependent on clinical trials. The application of the above techniques can predict drug targets, predict ligand–target interactions at the molecular level, and simulate the ligand–target time evolution process, reducing the large number of screen processes^24–26^. Therefore, this study used network pharmacology, molecular docking, and molecular dynamics simulation methods to analyze the key targets and related mechanisms of ellagic acid against RV, to evaluate the potential of ellagic acid as an anti-RV drug. Finally, SPR was used to verify the anti-RV target of ellagic acid.

## Results

### Target screening of ellagic acid against RV

**Figure 1:**
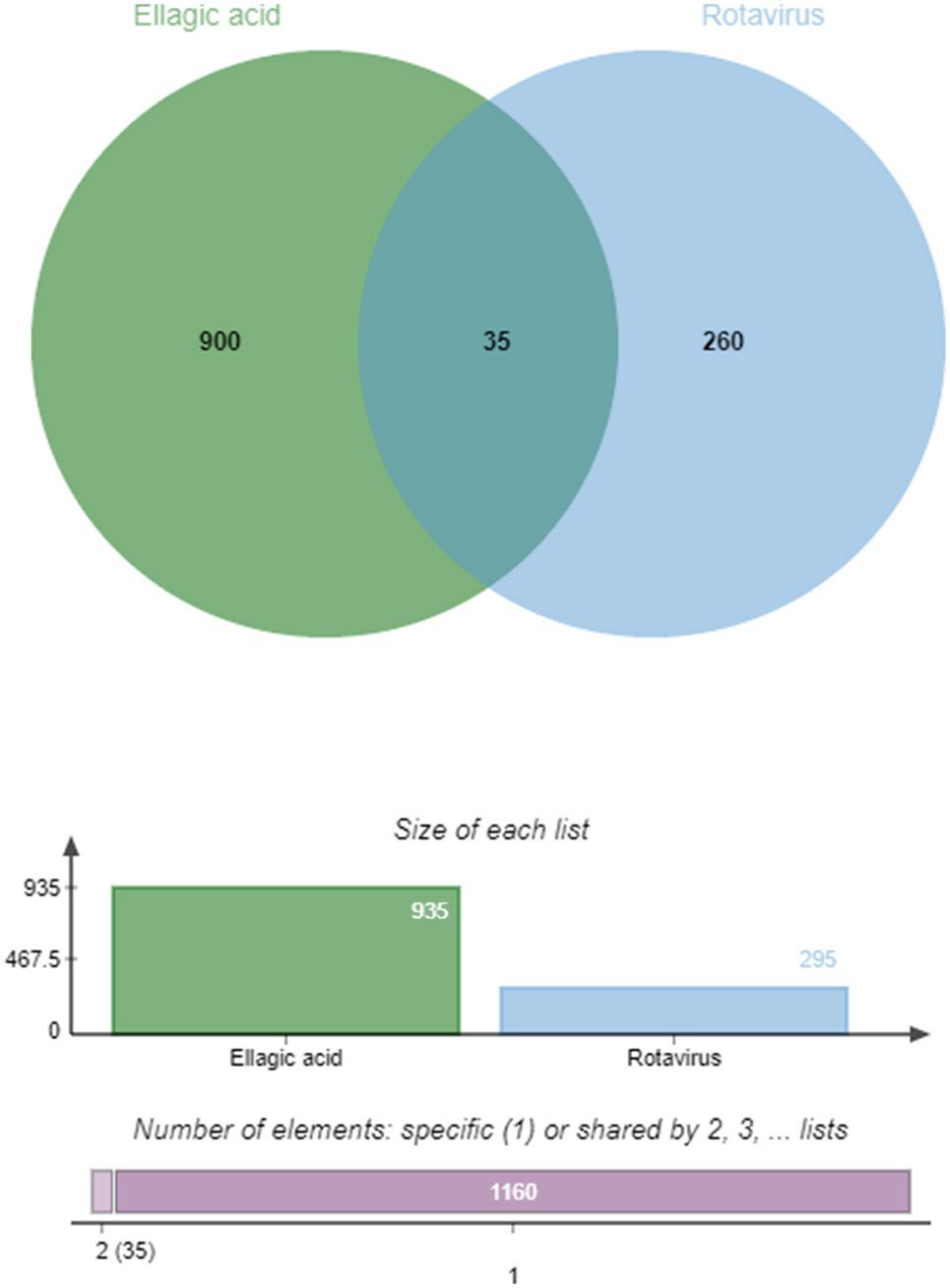
Venn diagram of potential targets of ellagic acid anti-RV.

935 ellagic acid targets were collected through 11 databases such as HIT, TCMSP, and BATMAN, and 295 anti-RV targets were collected through the Gene Cards database, and the target species information was human. The targets collected above were intersected, and the obtained protein was the potential target for ellagic acid to exert RV resistance. The target for ellagic acid to exert RV resistance was visualized by the Venn diagram, and the results are shown in Fig. 1. There were a total of 35 intersection proteins, and the results are shown in Table 1.

**Table 1:** The target of ellagic acid anti-RV.

Serial number	Gene	Uniprot ID	Name
1	TLR4	O00206	Toll-like receptor 4
2	TNF-α	P01375	Tumor necrosis factor
3	TP53	P04637	Tumor protein p53
4	IL-1β	P18510	Interleukin-1β
5	ALB	P02768	Albumin
6	NF-κB1	P19838	Nuclear factor-kappa B
7	CCL2	P13500	C–C motif chemokine ligand 2
8	TLR2	O60603	Toll-like receptor 2
9	MMP2	P08253	Matrix metalloproteinases 2
10	SMAD3	P84022	SMAD family member 3
11	ATM	Q13315	Ataxia telangiectasia-mutated
12	REN	P00797	Renin
13	BRAF	P15056	Serine/Threonine kinase proteins braf
14	LTF	P02788	Lactotransferrin
15	ACE2	Q9BYF1	Angiotensin-converting enzyme 2
16	CASP3	P42574	Caspase-3
17	MMP9	P14780	Matrix metalloproteinase-9
18	IL-4	P24394	Interleukin-4
19	TGF-β	P01137	Transforming growth factor β
20	CXCL8	P10145	Interleukin-8
21	EGF	P01133	Epidermal growth factor
22	IL-2	P60568	Interleukin-2
23	TTR	P02766	Transthyretin
24	BMP6	P22004	Bone morphogenetic protein 6
25	PML	P29590	Promyelocytic leukemia
26	EIF4G1	Q04637	eukaryotic initiation factor 4 γ1
27	TF	P02787	Transferrin
28	HRH2	P25021	Histamine receptor H2
29	PI3KR1	P27986	PI 3 kinase p85 alpha
30	HSPA8	P11142	Heat shock protein A8
31	PRKCA	P17252	Protein kinase C alpha
32	ITGA4	P13612	Integrin alpha 4
33	CGAS	Q8N884	Cyclic GMP-AMP synthase
34	HSPA1A	P0DMV8	Heat shock protein A1A
35	ACAD8	Q9UKU7	Acyl-CoA dehydrogenase 8

### PPI network

**Figure 2:**
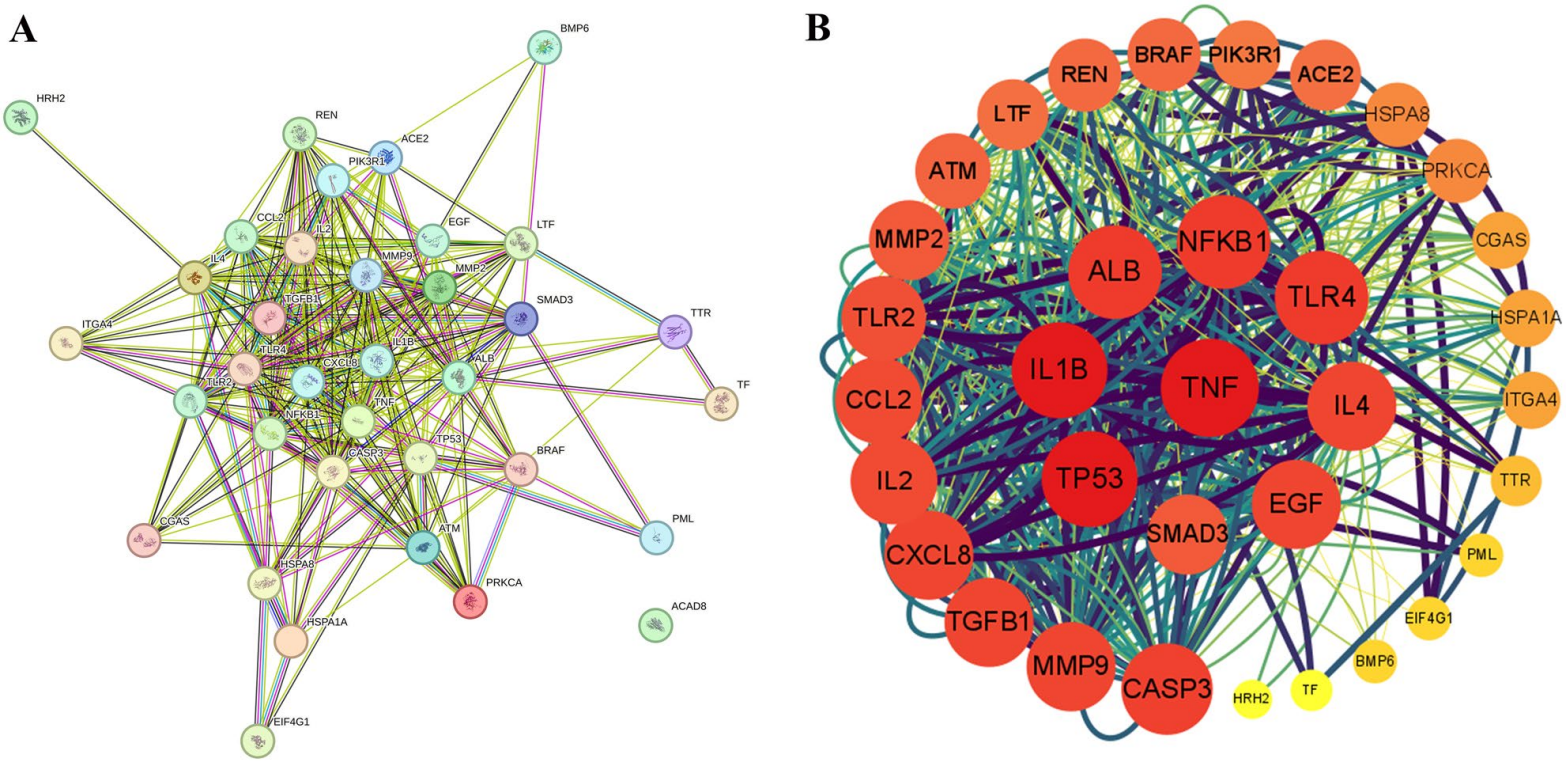
Ellagic acid potential RV targets the PPI network (**A**) and Hithubs network (**B**).

35 intersection targets were imported into the STRING database to obtain the protein–protein interaction network diagram (Fig. 2A). The tsv files of intersection targets were imported into Cytoscape for visualization, analysis, and draw the Hithubs network (Fig. 2B). The values of degree centralities, betweenness centralities, and closeness centralities among targets were obtained by CytoNCA plug-in. 9 core targets higher than the three average values (18.41, 0.67, 32.82) were screened based on the average values of betweenness centralities, closeness centralities and degree centralities. Results showed that the core targets of ellagic acid anti-RV were IL-1β, ALB, NF-Κb, TLR4, TNF-α, TP53, SAMD3, EGF, and IL-4. The screening process and results were shown in Supplementary File S1.

### Enrichment analysis

**Figure 3:**
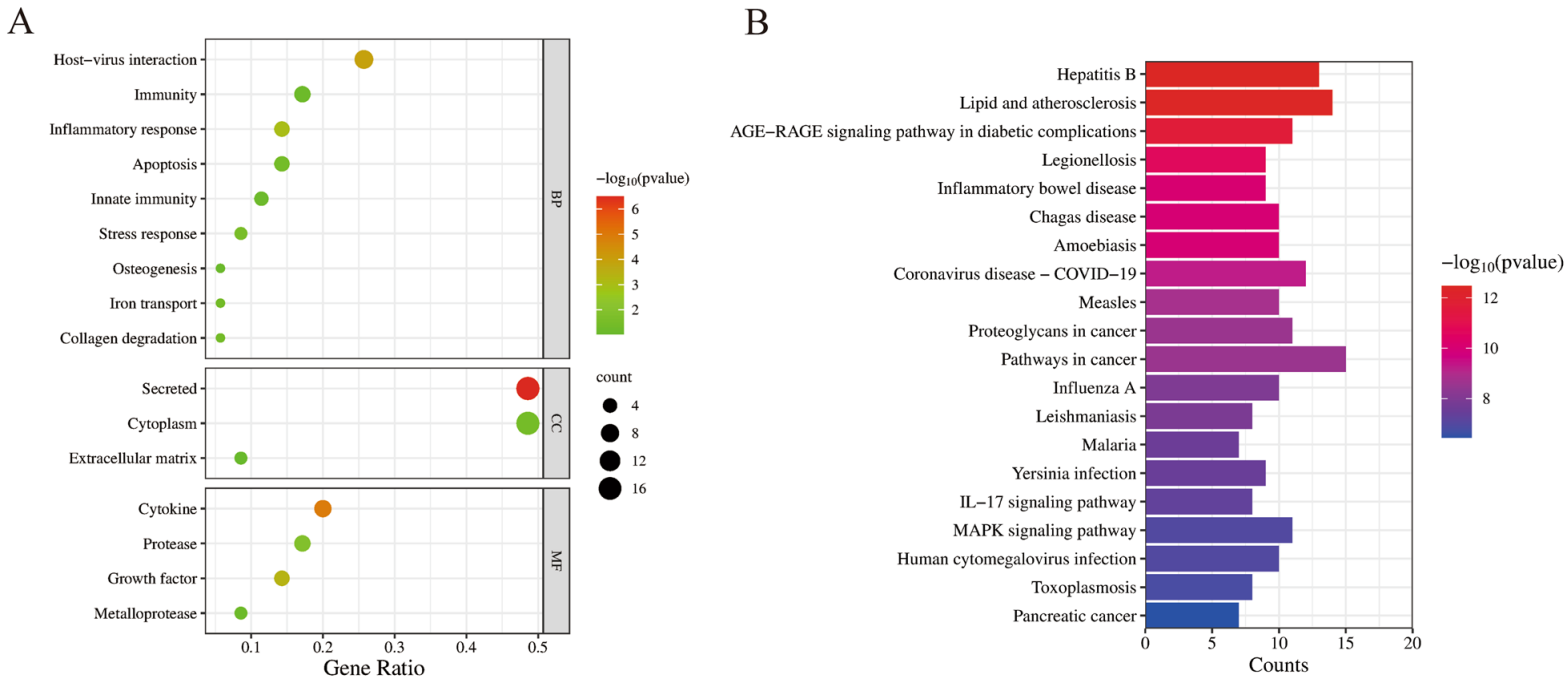
Construction of ellagic acid–target–pathway network. (**A**) The target that was enriched by the Gene Ontology of ellagic acid anti-RV. (**B**) The target that was enriched by the KEGG pathway of ellagic acid anti-RV.

A total of 35 ellagic acid anti-rotavirus targets were input into the DAVID database for GO and KEGG enrichment analysis, the results showed that 16 GO terms (including 9 BP, 3 CC, and 4 MF) and 122 KEGG pathways were enriched, the results are shown in Supplementary File S2. The enriched GO terms and KEGG pathways was sorted in descending order according to *P* value, a smaller *P* value represents a higher reliability of enrichment, the top 20 terms and pathways in *P* values were visualized in Fig. 3. In this study, the results showed that the targets of EA anti-RV were mainly enriched in biological processes such as host-virus interaction, inflammatory response, stress, and immunity; cellular components such as secretion, cytoplasm, and extracellular matrix; molecular functions such as cytokines, growth factors, proteinases, and metalloproteinases (Fig. 3A). Host-virus interaction (*P* value = 1.22E−4), Secreted (*P* value = 3.12E−7), and Cytokine (*P* value = 1.15E−5) are the most relevant enriched terms of biological processes, cellular components, and molecular functions, respectively. The targets of EA anti-RV were significantly enriched in KEGG pathways such as inflammatory bowel disease, IL-17 signaling pathway, and MAPK signaling pathway (Fig. 3B). The results showed that the anti-RV effect of ellagic acid may be related to the regulation of cellular inflammatory response.

### Construction of ellagic acid-target-pathway network

**Figure 4:**
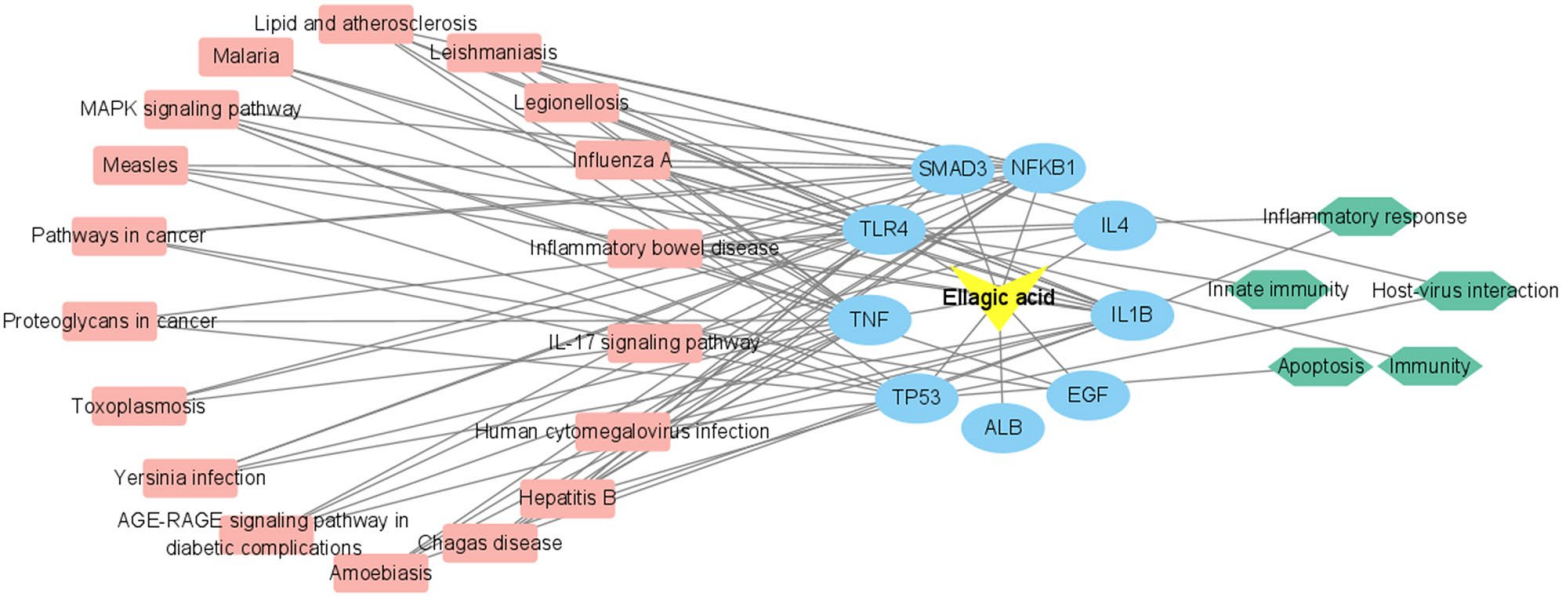
Ellagic acid–target–pathway network model.

The 9 core targets of EA anti-RV were integrated with the functions and pathways enriched in the top 20 *P* values, and the ellagic acid–target–pathway–function network was constructed by Cyotoscape (Fig. 4). Nodes in the network diagram represent EA, target protein, enrichment function, and pathway respectively. The side represents the interaction between the EA and the corresponding functions and pathways of target proteins, and there were 87 pairs of pathway–protein–function relationships, including 8 pairs of protein–function relationships and 79 pairs of pathway–protein relationships. The results were shown in Supplementary File S3. The network diagram showed that EA can play antiviral, anti-inflammatory, and immunomodulatory roles by regulating pathway-related target proteins.

### Molecular docking analysis

**Figure 5:**
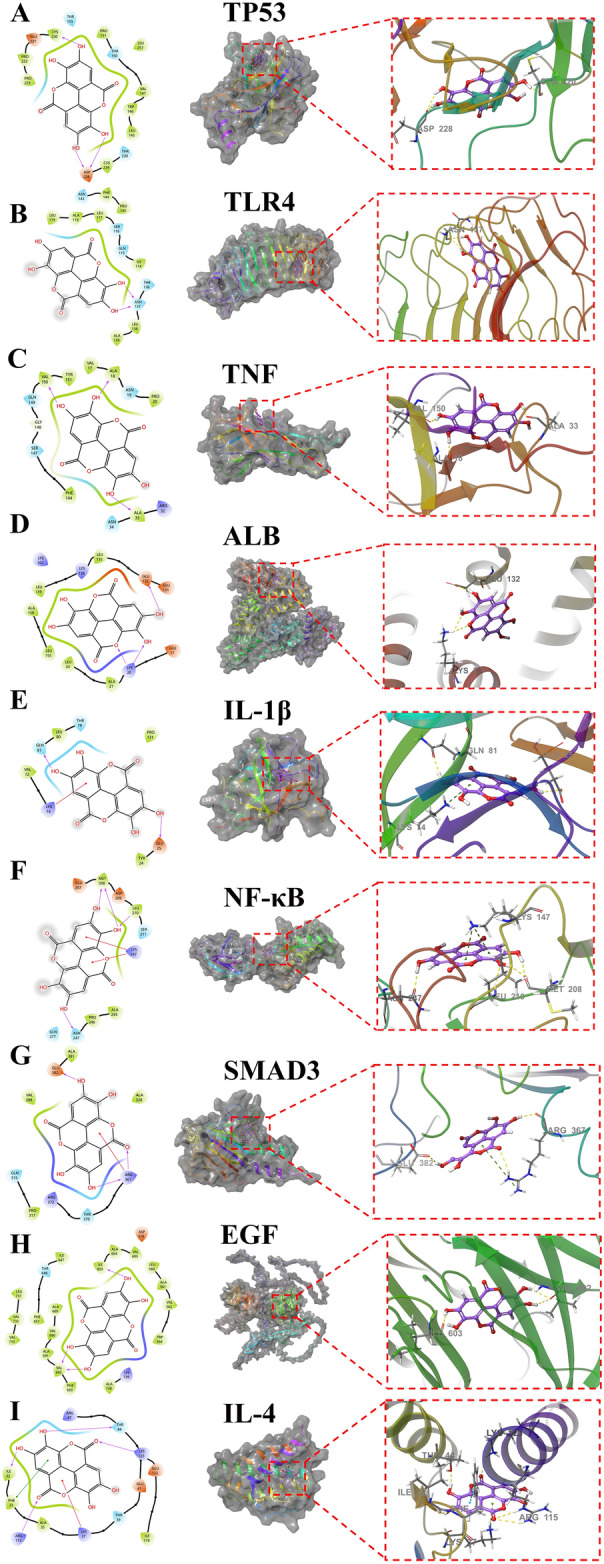
Molecular docking complexes of ellagic acid with TP53 (**A**), TLR4 (**B**), TNF (**C**), IL-1β (**D**), NSP5 (**E**), NF-κB (**F**), SAMD3C (**G**), EGFC (**H**) and IL-4C (**I**) (yellow represents hydrogen bonds, and green represents π-Cation bonds).

EA was paired to the surface of TP53, TLR4, TNF-α, ALB, IL-1β, NF-κB1, SAMD3, EGF, and IL-4 active pockets, respectively. EA formed hydrophobic forces with residues such as CYS229, LEU145, TRP146, VAL147 and PRO151 of TP53, and formed one hydrogen bond with residue CYS220 and two hydrogen bonds with residue ASP228 (Fig. 5A). EA formed hydrophobic forces with residues ALA139, LEU138, ILE114, LEU117, ALA118 of TLR4, and formed two hydrogen bonds with residues ASN137 (Fig. 5B). EA formed hydrophobic forces with residues such as PRO20, ALA18, VAL17, TYR151, and VAL150 of TNF-α, and formed hydrogen bonds with residues such as ALA33, VAL150, and ALA18 (Fig. 5C). EA formed hydrophobic forces with residues LEU24, LEU155, ALA158, LEU139, LEU135 of ALB, and formed a hydrogen bond with residues GLU132 and two hydrogen bonds with residues LYS20 (Fig. 5D). EA formed a hydrogen bond with GLN81 and GLU25 residues of IL1β, and a π-Cation (π-cation) bond with residues LYS74 (Fig. 5E). EA formed hydrophobic forces with residues MET208 and LEU210 of NFKB1, formed one hydrogen bond with residues ASN247 and LEU210, two hydrogen bonds with residues MET208, and two π-Cation (π-cation) bonds with residues LYS147 (Fig. 5F). EA formed hydrophobic forces with residues ILE32, PHE33 and ALA35 of IL4, formed a hydrogen bond with residues ILE32, THR44 and LYS123, formed two hydrogen bonds with residues ARG115, and forms a π-Cation (π-cation) bond with residues LYS37. Form a π–π bond with residue PHE33 (Fig. 5G). EA formed hydrophobic forces with residues such as VAL388, ALA381, and ALA328 of SMAD3, forming two hydrogen bonds and a π-Cation bond with residue ARG367, and a hydrogen bond with residue GLU382 (Fig. 5H). EA formed hydrophobic forces with residues such as ALA691, VAL690, ALA689, ILE603, and ALA604 of EGF, forming a hydrogen bond with residues ILE603 and two hydrogen bonds with residues VAL692 (Fig. 5I).

**Figure 6:**
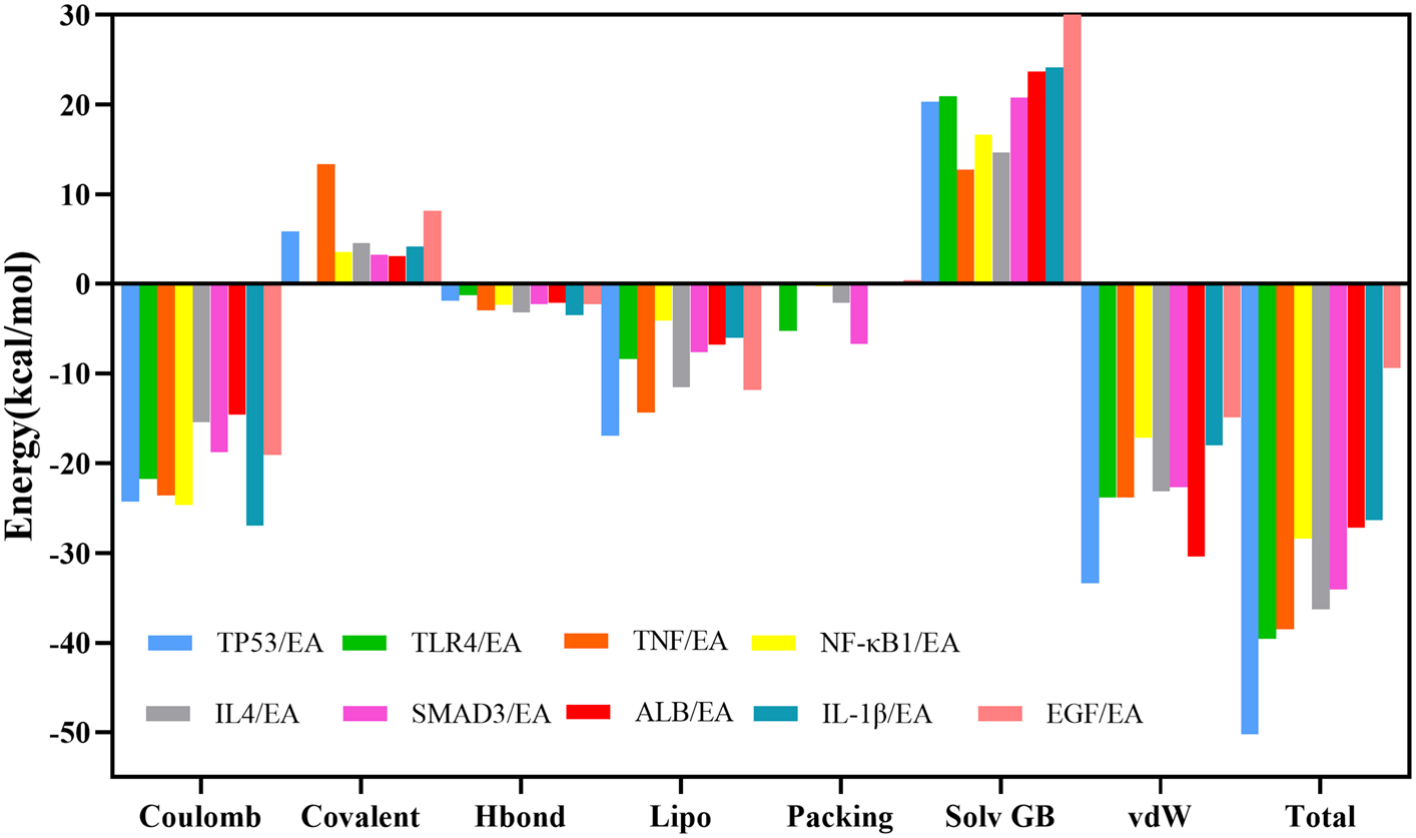
Statistical diagram of the MM/GBSA calculation for the complexes.

As shown in the results of XP (Table 2) and MM-GBSA energy (Table 3, Fig. 6), the score of EA with TP53 and TLR4 were − 9.115 and − 6.285, respectively, and the results of MM-GBSA energy were − 50.22 kcal/mol and − 39.58 kcal/mol, respectively. The low binding free energy and docking scores indicate that EA has strong binding stability with TP53 and TLR4. The binding free energy of EA with TNF-α, IL-4, and SAMD3 was lower than − 30 kcal/mol, but the docking score was higher than − 6, indicating that the binding stability of EA with TNF-α, IL-4, and SAMD3 was good. In addition, EA had higher docking scores or binding free energies with IL1-β, NFKB1, ALB, and EGF, indicating that the binding of EA to these four proteins was unstable.

**Table 2:** XP docking score of the core target with ellagic acid.

Compound	Target	XP Gscore
Ellagic acid	TP53	− 9.115
	EGF	− 7.031
	TLR4	− 6.285
	TNF-α	− 5.501
	ALB	− 5.249
	IL-4	− 5.16
	IL-1β	− 4.276
	SAMD3	− 3.901
	NF-κB1	− 3.709

**Table 3:** Statistical analysis of MM/GBSA results.

Energy	TP53	TLR4	TNF-α	IL-4	SMAD3	NF-κB1	ALB	IL-1β	EGF
	Ellagic acid								
Coulomb	− 24.23	− 21.76	− 23.53	− 15.42	− 18.77	− 24.64	− 14.57	− 26.94	− 19.02
Covalent	5.86	0.06	13.35	4.54	3.26	3.58	3.08	4.18	8.15
Hbond	− 1.93	− 1.30	− 2.97	− 3.20	− 2.25	− 2.36	− 2.15	− 3.50	− 2.27
Lipo	− 16.87	− 8.41	− 14.37	− 11.57	− 7.67	− 4.11	− 6.81	− 6.01	− 11.83
Packing	− 0.00	− 5.24	− 0.00	− 2.16	− 6.74	− 0.31	0.00	− 0.20	− 0.08
Solv GB	20.32	20.90	12.77	14.66	20.75	16.64	23.66	24.16	30.52
vdW	− 33.36	− 23.83	− 23.78	− 23.12	− 22.63	− 17.17	− 30.36	− 18.00	− 14.89
Total	− 50.22	− 39.58	− 38.53	− 36.28	− 34.05	− 28.38	− 27.15	− 26.3	− 9.42

### MD simulations

**Figure 7:**
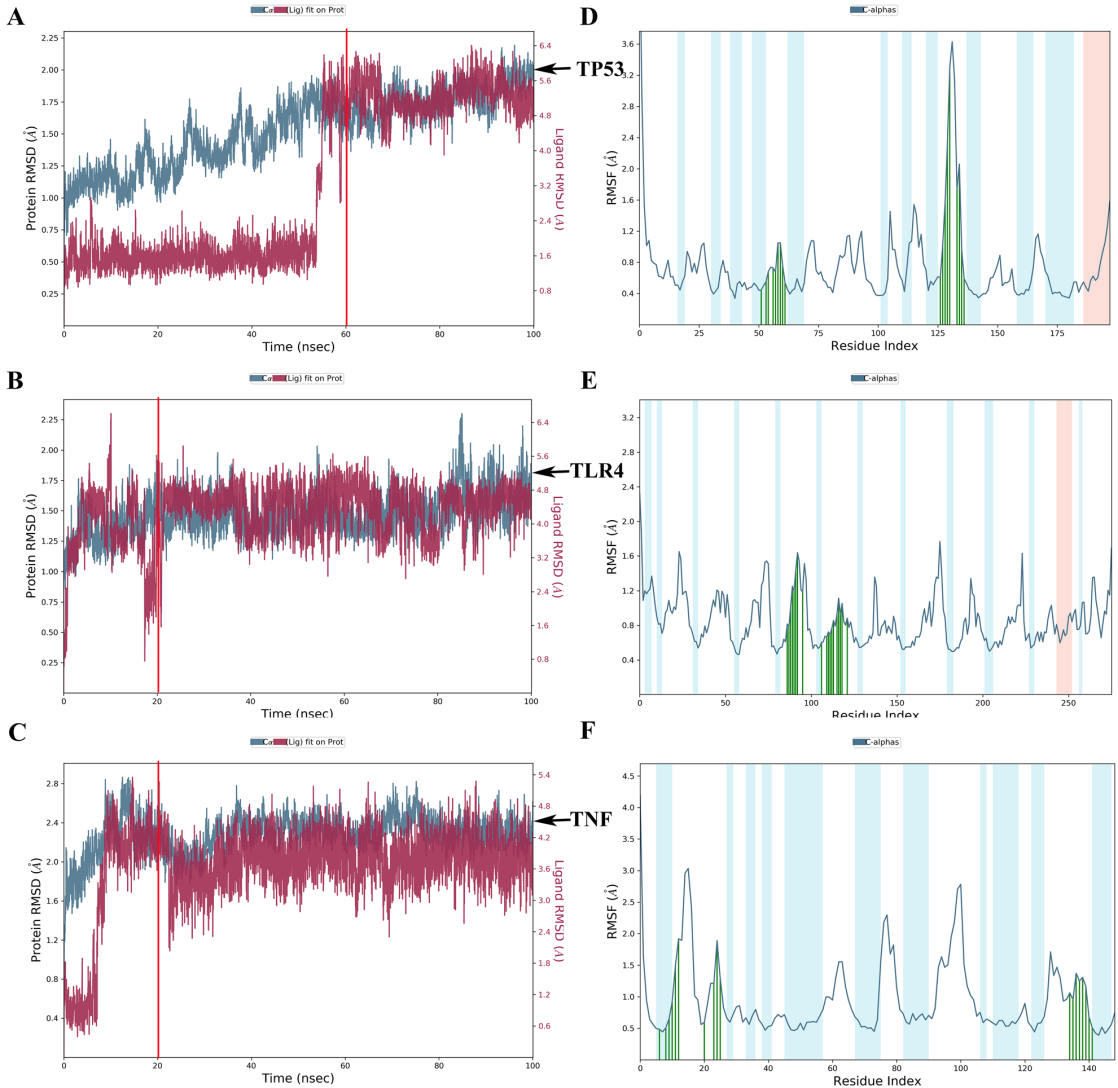
RMSD and RMSF plots throughout the 100 ns MD simulation. (**A**–**C**) The molecular dynamics simulation -RMSD value (the blue line represents the proteins, and the red line represents ellagic acid). (**D**–**F**) The molecular dynamics simulation -RMSF value (α-helical and β-strand regions are highlighted in red and blue backgrounds, respectively. Protein residues that interact with the ligand are marked with green-colored vertical bars).

EA and TP53, TLR4, and TNF-α proteins were simulated by 100 ns MD, and their molecular dynamics trajectories were analyzed. The results showed that EA and TP53, TLR4, and TNF proteins were stable after 60, 20, and 20 ns, respectively, and the system was in a state of equilibrium (Fig. 7A–C). When EA bound to TP53, the protein showed high structural flexibility in the 125–130 AA residue region (Fig. 7D). After binding to TLR4 protein, EA showed high structural flexibility in the 20–30 AA, 60–80 AA, and 90–100 AA residue regions (Fig. 7E). When EA bound to TNF-α protein, the protein exhibited high structural flexibility in the 10–20 AA, 75–85 AA, and 90–105 AA residue regions (Fig. 7F).

**Figure 8:**
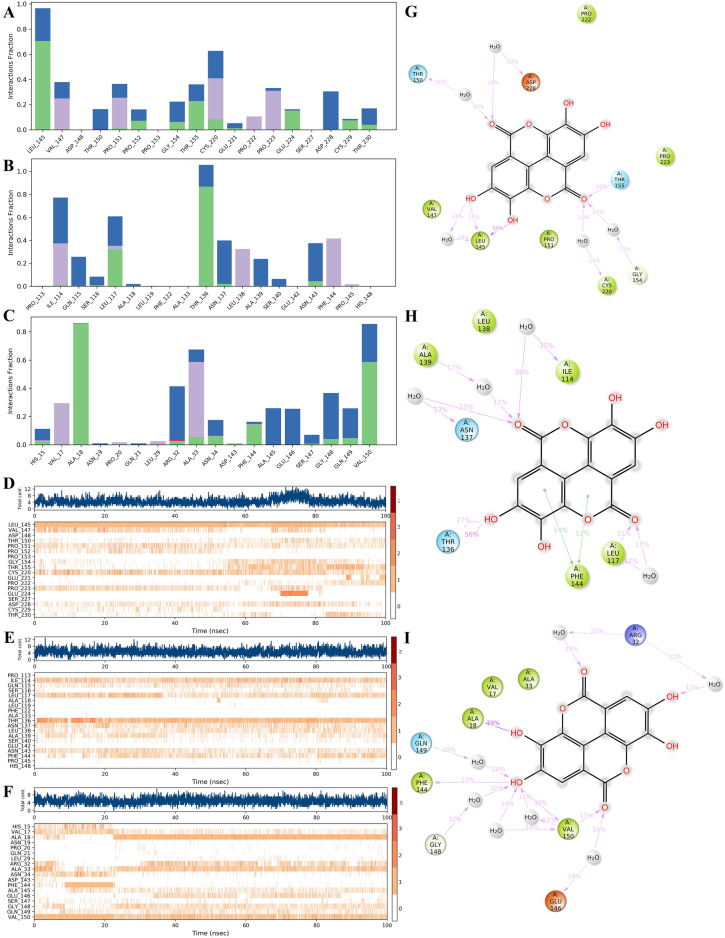
The interaction and binding mode of ellagic acid with TP53, TLR4, and TNF protein in molecular dynamics simulation. (**A**–**C**) The contribution of amino acids at TP53, TLR4, and TNF binding sites to EA-protein binding, respectively. (**D**–**F**) How interactions between EA and specific amino acids of the TP53, TLR4, and TNF proteins have changed over time, respectively (shown in orange with varying depths, according to the proportions on the right side of the figure). (**G**–**I**) A detailed diagram of EA's interactions with TP53, TLR4, and TNF protein residues.

Protein–ligand interactions can be monitored throughout the simulation. The main amino acids that play an important role in the binding of EA and TP53 protein are LEU145, VAL147, and CYS220, and their interactions are mainly water bridge, hydrogen bond, and hydrophobic action (Fig. 8A). Amino acids that play an important role in the binding of EA to TLR4 protein are ILE114, LEU117, and THR136, and their interactions are mainly water bridge, hydrogen bond, and hydrophobic action (Fig. 8B). The amino acids that play an important role in the binding of EA to TNF-α protein are mainly ALA18, ARG32, ALA33, and VAL150, and their interactions are mainly water bridge, hydrogen bond, and hydrophobic action (Fig. 8C).

During the entire trajectory, the interaction between EA and specific amino acids of TP53, TLR4, and TNF-α proteins, respectively changed over time as shown in Fig. 8D–F. The results showed that TP53 amino acid residues LEU145, VAL147, PRO151, THR155, CYS220, PRO223, and ASP228 had multiple contacts with EA (Fig. 8D). TLR4 amino acid residues ILE114, GLN115, LEU117, THR136, ASN137, LEU138, ALA139, ASN143, and PHE144 had multiple contacts with EA (Fig. 8E). TNF-α amino acid residues VAL17, ALA18, ARG32, ALA33, SAN34, PHE144, ALA145, GLU146, GLY148, GLN149, and VAL150 had multiple contacts with EA (Fig. 8F). The diagram is shown in Fig. 8G–I. The conformational evolution of each RB in ellagic acid within the entire simulated locus (0–100 ns) is shown in Supplementary Figure S1.

### Key binding sites saturated mutation affinity results

**Figure 9:**
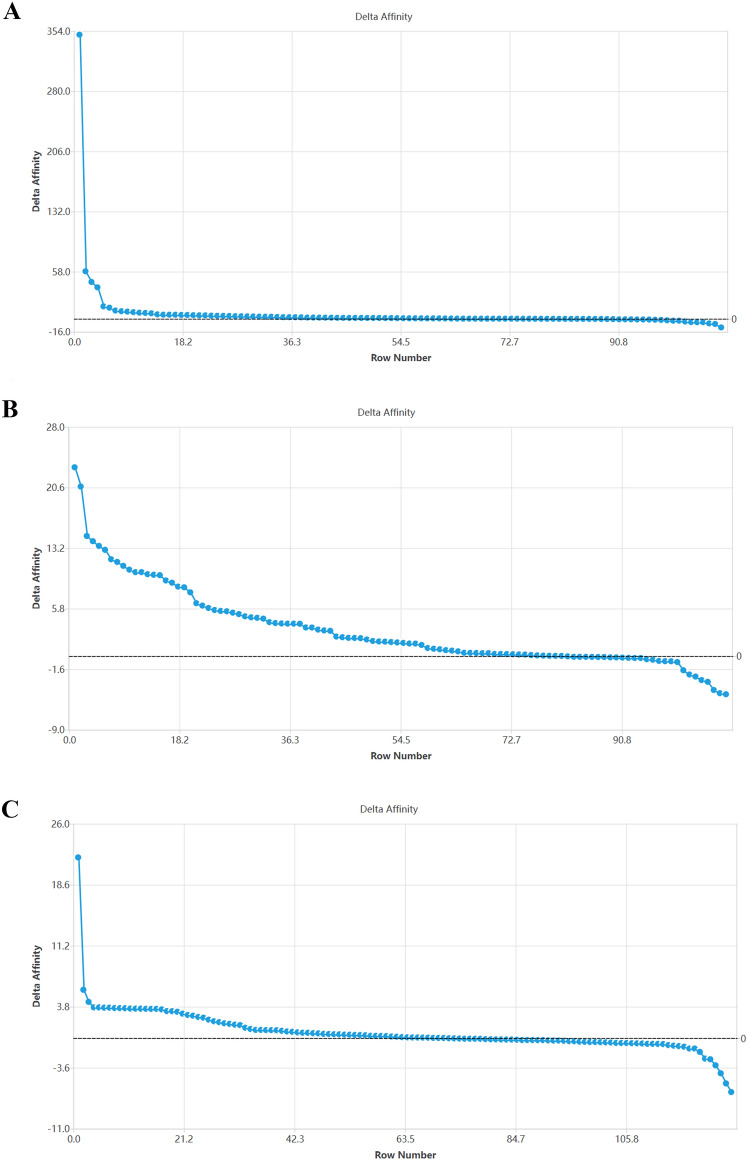
Trend diagram of saturation mutagenesis results at key binding sites.

After saturation mutation of key binding sites, the Δaffinity results of TP53 were 220 (CYS → TRP), 220 (CYS → TYR), and 220 (CYS → PHE), respectively. The corresponding values were 349.066 kcal/mol, 58 kcal/mol and 44.884 kcal/mol, respectively (Fig. 9A). The highest Δaffinity results of TLR4 were 136 (THR → TYR), 136 (THR → PHE), and 136 (THR → TRP), corresponding to 23.015 kcal/mol, 20.665 kcal/mol, and 14.626 kcal/mol (Fig. 9B). The highest Δaffinity results for TNF-α were 150 (VAL → TRP), 18 (ALA → GLU), and 144 (PHE → GLY), corresponding to 21.868 kcal/mol, 5.812 kcal/mol, and 4.338 kcal/mol (Fig. 9C).

**Figure 10:**
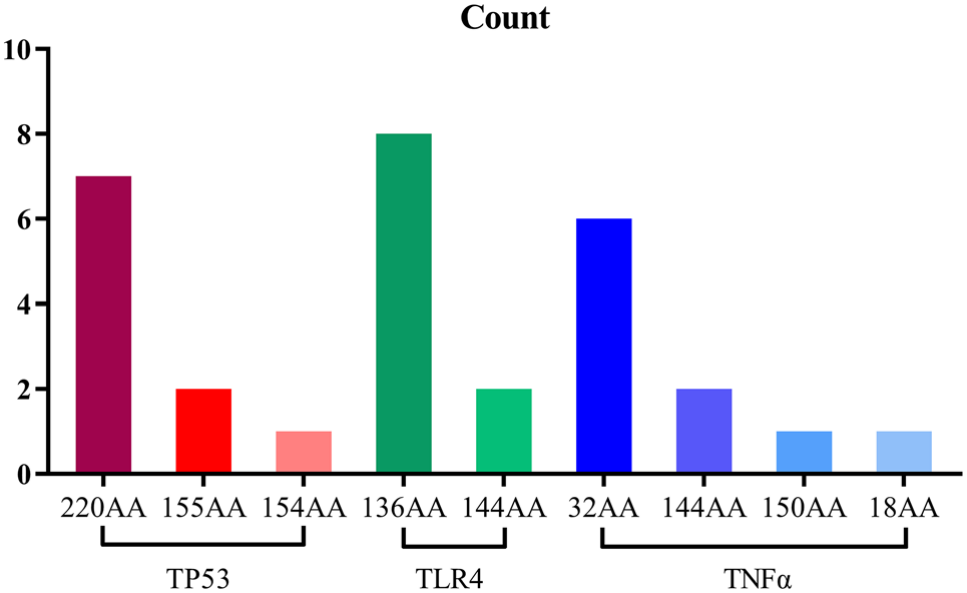
TOP10 results of saturation mutagenesis affinity of key binding sites.

Among the top10 Δaffinity results of TP53, TLR4, and TNF-α (Table 4), TP53 occurred more frequently at 220 sites, TLR4 occurred more frequently at 136 and 144 sites, and TNF-α occurred more frequently at 32 and 144 sites, respectively (Fig. 10). The MM-GBSA binding free energy results of affinity mutations at the top 10 Δaffinity results of TP53, TLR4, and TNF-α are shown in Table 5. These mutations inhibit binding by weakening the ligand's van der Waals force on the protein. It is difficult to find significant changes in the two-dimensional structure of the top3 sites with Δaffinity of TP53, TLR4, and TNF-α, which indicates that the mutation mainly affects non-covalent bonds such as hydrogen bonds and salt bridges (Supplementary Fig. S2).

**Table 4:** TOP10 saturation mutagenesis affinity of key binding sites.

TP53		TLR4		TNF-α	
Mutations	△Affinity(kcal/mol)	Mutations	△Affinity(kcal/mol)	Mutations	△Affinity(kcal/mol)
A:220 (CYS → TRP)	349.066	A:136 (THR → TYR)	23.015	A:150 (VAL → TRP)	21.868
A:220 (CYS → TYR)	58.000	A:136 (THR → PHE)	20.665	A:18 (ALA → GLU)	5.812
A:220 (CYS → PHE)	44.884	A:136 (THR → TRP)	14.626	A:144 (PHE → GLY)	4.338
A:220 (CYS → MET)	38.254	A:144 (PHE → GLY)	13.993	A:32 (ARG → GLY)	3.667
A:220 (CYS → ASP)	14.819	A:136 (THR → ARG)	13.405	A:32 (ARG → TYR)	3.665
A:220 (CYS → GLU)	13.185	A:136 (THR → GLU)	12.941	A:144 (PHE → LYS)	3.645
A:155 (THR → GLN)	9.713	A:136 (THR → LYS)	11.780	A:32 (ARG → TRP)	3.640
A:155 (THR → MET)	8.879	A:136 (THR → ASN)	11.461	A:32 (ARG → GLU)	3.588
A:220 (CYS → THR)	8.409	A:144 (PHE → ALA)	10.981	A:32 (ARG → ASP)	3.577
A:154 (GLY → ARG)	7.747	A:136 (THR → ILE)	10.509	A:32 (ARG → SER)	3.573

**Table 5:** The change of MM-GBSA binding free energy in saturation mutagenesis affinity TOP3 at key binding sites.

Protein	Title	Δaffinity	Coulomb	Hbond	Lipo	Packing	Solv GB	vdW
TP53	A:220 (CYS → TRP)	349.066	3.572	− 0.003	− 6.662	− 8.580	0.152	360.587
	A:220 (CYS → TYR)	58.000	4.430	0.000	− 3.737	− 3.739	2.639	58.408
	A:220 (CYS → PHE)	44.884	4.139	0.000	− 3.671	− 2.883	1.273	46.025
TLR4	A:136 (THR → TYR)	23.015	6.967	0.244	− 0.914	− 0.005	− 0.658	17.381
	A:136 (THR → PHE)	20.665	4.634	0.250	− 1.565	0.000	− 2.176	19.523
	A:136 (THR → TRP)	14.626	7.292	0.248	− 1.140	0.000	− 0.037	8.263
TNF-α	A:150 (VAL → TRP)	21.868	− 2.707	− 0.069	− 1.257	0.000	− 1.616	27.517
	A:18 (ALA → GLU)	5.812	0.714	− 0.019	− 0.230	0.000	6.295	− 0.947
	A:144 (PHE → GLY)	4.338	− 0.159	0.000	2.215	0.001	0.370	1.911

### The affinity between ellagic acid and TLR4 protein

**Figure 11:**
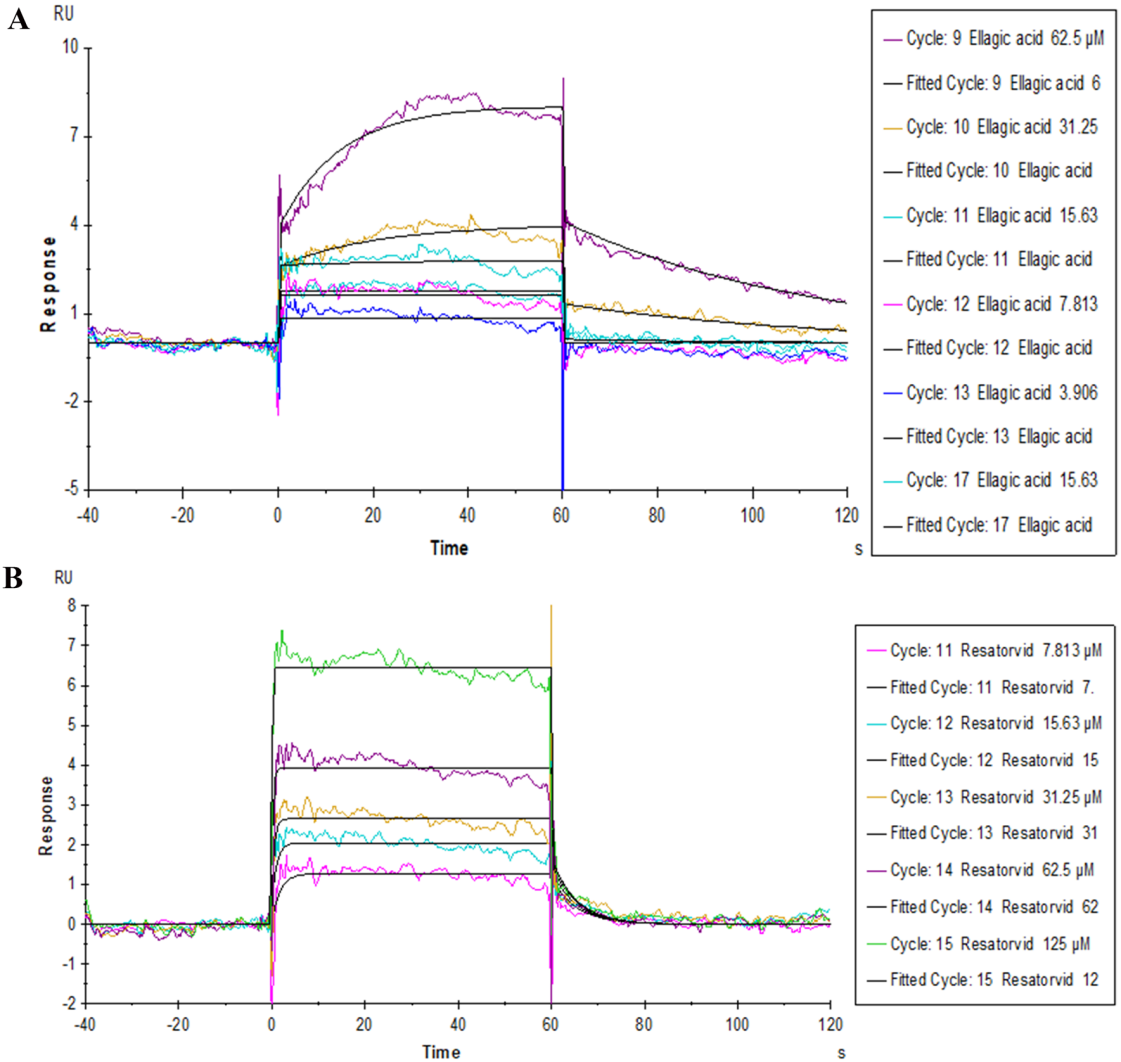
Surface plasmon resonance was used to test the affinity of TLR4 protein with different concentrations of ellagic acid (**A**) and resatorvid (**B**).

SPR biosensor was used to detect the affinity between ellagic acid and resatorvid with TLR4 protein. The results showed that the dissociation constant Kd value of ellagic acid and resatorvid was low, and the affinity parameters are shown in Table 6. Both ellagic acid (Fig. 11A) and resatorvid (Fig. 11B) had a good affinity for the fitting curves produced by reacting with TLR4 protein.

**Table 6:** Affinity of TLR4 protein with ellagic acid and resatorvid.

Protein	Ligand	KD (M)	Rmax (RU)
TLR4	Resatorvid	3.725 × 10^–6^	1.700
	Ellagic acid	1.917 × 10^–5^	8.000

## Discussion

Pomegranate peel is often used in TCM to treat diarrhea, and its specific compound is ellagic acid, so it has a very high anti-RV potential value^13^. This study used network pharmacology, molecular docking, and molecular dynamics simulations to evaluate ellagic acid's anti-RV activity and potential mechanisms. In this study, a total number of 35 anti-RV targets of ellagic acid were discovered through network pharmacology, and 9 core targets (TP53, TLR4, TNF-α, ALB, IL-1β, NF-κB, IL-4, SAMD4, and EGF) were screened by constructing PPI networks.

Molecular docking is a technique that is widely used in drug development based on computer structure. Among them, the XP mode is flexible docking (both protein and ligand are flexible), and it is also the most detailed calculation mode that can be used for molecular docking calculation with higher resolution for specific targets^27^. XP docking results need to refer to XP Gscore, which is generally believed to be less than − 6, indicating that the ligand and protein have stable binding properties. The value of MM-GBSA energy is lower than − 30 kcal/mol, indicating that the ligand binds to the protein stably^28^. The scores of EA docking with TP53 and TLR4 are − 9.115 and − 6.285 respectively, which are less than − 6. The results of MM-GBSA energy were − 50.22 and − 39.58 kcal/mol, which were lower than − 30 kcal/mol. The results showed that EA was strongly bound to TP53 and TLR4. The binding free energy of EA with TNF-α, IL-4, and SAMD3 was lower than − 30 kcal/mol, but the docking score was higher than − 6, indicating that EA was well bound with TNF-α, IL-4, and SAMD3. However, the docking scores or MM-GBSA energy of EA with IL1-β, NFKB1, ALB, and EGF were lower, indicating that the binding of EA to these four proteins was unstable. Therefore, this study narrowed the range of EA anti-RV targets to TP53 and TLR4. In molecular docking experiments, MM-GBSA energy usually has a higher correlation between experimental results and screening ability than molecular docking scores, so this study also selected TNF-α, which has low MM-GBSA energy with EA.

Rotavirus infection can activate NF-κB, leading to severe diarrhea, and NF-κB and TP53 can interfere with each other through different mechanisms to affect its activity^29^. For example, NF-κB can up-regulate the expression of TP53 inhibitor MDM2^30^. IKKβ activates NF-κB through phosphorylation of IκB and also reduces the stability of p53 through its post-translational modification^31^. TP53 is one of the glucocorticoid receptors, and inactive TP53 leads to impaired anti-inflammatory function of glucocorticoids^32^. Ellagic acid can promote the expression of TP53, so its anti-RV mechanism may antagonize NF-κB activity and inhibit the generation of inflammation by promoting the expression of TP53^33^. However, TP53, as an antioncogene, is mainly involved in cancer-related processes, so the above pathways may be second.

TNF-α mainly produced by activated macrophages, is a potent pro-inflammatory cytokine that plays a crucial role in the regulation of immunity, inflammation, cell growth, differentiation, and apoptosis^34^. TNF-α can activate the IKK complex (IKK-α, IKk-β, and IKk-γ) by binding to the TNF receptor (TNFR). Induction of phosphorylation and degradation of IκB, activation of NF-κB, and nuclear translocation, ultimately lead to inflammation^35^. Liu et al. found that the serum TNF-α content in children with RV enteritis increased significantly, indicating that TNF-α was involved in the process of RV enteritis^36^. However, Hakim et al. found that TNF-α exerts an anti-RV effect by activating the classical NF-κB pathway, and this opposite result may depend on the degree of rotavirus infection^37^. Therefore, EA may interfere with the NF-κB signaling pathway by binding to TNF-α, inhibiting RV replication. The amino acids that play an important role in the binding of EA to TNF-α protein are mainly ALA18, ARG32, ALA33, and VAL150, and their interactions are mainly hydrobridging, hydrogen bonding, and hydrophobic. The ΔAffinity score is greater than 0 indicating that the affinity between the mutant protein and the ligand is lower than that between the original protein and the ligand indicating that the affinity is weakened and that the mutant amino acid site is the key to protein–ligand binding^28^. The highest Δaffinity results for TNF-α were 150 (VAL → TRP), 18 (ALA → GLU), and 144 (PHE → GLY), corresponding to 21.868 kcal/mol, 5.812 kcal/mol, and 4.338 kcal/mol respectively, and the Δaffinity scores were all greater than 0. It shows that these three sites play a key role in protein–ligand binding and these amino acid sites are distributed in the cytoplasmic, transmembrane, and extracellular domains of TNF-α (according to the Uniprot database). Drug targets are generally receptors, enzymes, ion channels, and nucleic acids. TNF-α is essentially an inflammatory cytokine that plays a pro-inflammatory role, but in infection-induced diarrhea, IL-6, IL-1β, and other inflammatory cytokines have similar roles^38^. Therefore, it is incomplete to consider TNF-α as a potential anti-RV target.

Toll-like receptors (TLRs) as transmembrane inflammatory receptors, participate in mucosal innate immune regulation^39^. TLR4, a member of the TLRs family recognizes LPS and is localized to the cell membrane and cytoplasm^40^. TLR4 can also recognize glycoproteins of a variety of viruses, such as the envelope protein (Env) of mouse mammary tumor virus (MMTV)^41^. Wang et al. found that RV-SA11 caused enteritis through TLR4/MyD88/ NF-κB signaling pathway^42^. Chen et al. found that RV infection of BALB/c mice can activate the TLR4/NF-κB signaling pathway^43^. The combination of TLR4-dependent Cludin-1 internalization and secretagogue-mediated chloride secretion leads to diarrhea^44^. A growing number of studies have focused on blocking the TLR4/NF-κB pathway for the treatment of gastrointestinal diseases, but whether TLR4 binds to RV protein is unclear. Amino acids that play an important role in the binding of EA to TLR4 protein are ILE114, LEU117, and THR136 and their interactions are mainly hydrobridge, hydrogen bond, and hydrophobic, which belong to the extracellular domain of TLR4 (23–631 AA)^45^. Studies have reported that TLR2 can recognize RV NSP4 protein to mediate the production of pro-inflammatory factors, so TLR4 may mediate the production of diarrhea by recognizing a certain RV protein^46^. Among the top 10 Δaffinity results of TLR4 protein, 136 and 144 sites appeared more frequently, which can be further verified in the future. SPR results showed that ellagic acid could bind TLR4 protein specifically. Combined with network pharmacology, computational biology, and SPR analysis, TLR4 can be used as a potential target for further study. Therefore, ellagic acid may inhibit the activation of the TLR4/NF-κB signaling pathway by binding to the extracellular domain of TLR4 and ultimately inhibiting RV replication.

## Materials and methods

### Compounds and reagents

CM5 Sensor Chip was purchased from Cytiva (USA). 1-Ethyl-3-(3-dimethylaminopropyl) carbodiimide (EDC), N-Hydroxysuccinimide (NHS), and ethanolamine were purchased from Sigma (USA). NaOH 50 mM and sodium acetate solution (pH4.0) were purchased from GE Healthcare (USA). Ellagic acid and resatorvid were purchased from MCE (USA), with 99.23% and 99.72% purity, respectively. Recombinant human TLR4 protein (Active) was purchased from Abcam (USA).

### Network pharmacology

#### Ellagic acid and rotavirus-related target screening

Based on previous work and related literature reports, this study used "Ellagic acid" and "Rotavirus" as search terms to collect relevant targets. Firstly, protein structure, Compound CID, MF, and SMILES of ellagic acid were obtained by downloading from the *PubChem* database (https://pubchem.nc-bi.nlm.nih.gov/). Obtained Ellagic Acid targets from the *HIT* (http://hit2.badd-cao.net/), *TCMSP* (https://old.tcmsp-e.com/), *BATMAN* (http://bionet.ncpsb.org.cn/batman-tcm/), *ITCM* (http://itcm.biotcm.net), *NPASS* (https://bidd.group/NPASS/), *TCMSID* (https://tcm.scbdd.com/home/index/), *HERB* (http://herb.ac.cn/), *SwissTargetPrediction* (http://swisstargetprediction.ch), *CHEMBL* (https://www.ebi.ac.uk/chembl/), *SymMap* (http://www.symmap.Org/) and *PharmMapper* (http://lilab-ecust.cn/pharmmapper/index.html) database. Finally, rotavirus-related target data were retrieved through the *Gene Cards* database (https://www.gene-cards.org).

#### Visual analysis of ellagic acid anti-rotavirus target and construction of PPI network

The screened ellagic acid-related targets were intersected with the rotavirus-related targets to map Venny. The data was imported into Venny platform (https://bioinfogp.cnb.csic.es/tools/Venny/index.HTML), the intersection targets were visualized, ellagic acid anti-rotavirus targets were collected. Then, the target was imported into the *STRING* database (https://string-db.org/), and the species ‘*homo–sapiens*’ was selected to obtain the interaction relationship of target proteins, and the protein–protein interaction network and tsv file were exported. Next, the tsv file was imported into Cytoscape v3.9.1 to draw the Hithubs network, Network Analyzer was used to conduct topological analysis on the network. Node size and color depth were used to reflect the degree score, edge thickness was used to reflect the combined score. The core of targets was screened according to the values of degree centralities, betweenness centralities, and closeness centralities.

#### GO enrichment and KEGG pathway analysis

Logged into the *DAVID* database (https://david.ncifcrf.gov/), imported ellagic acid anti-rotavirus potential targets, selected the species ‘*homo-sapiens*’, setted the identifier and list type to official gene symbol and gene list, respectively. GO function and KEGG pathway enrichment analysis were performed on potential ellagic acid anti-rotavirus targets, and the results were exported to a txt file. The targets were sorted according to *P* values, and the top 20 terms in *P* values that enriched by GO (Biological Process, Molecular Function, and Cellular Component) and KEGG were selected, and they were imported into the online platform (http://www.bioinformatics.com.cn/) draw column chart and bubble chart.

#### Construction of drug-target-pathway network

Log in to Cytoscape 3.9.1 software (https://cyto-scape.org/) to import the network file and type file of ellagic acid-core target-pathway, respectively, to construct the "drug-target-pathway" network diagram and beautify it with Layout and Style tools.

### Molecular docking

#### PDB ID (protein data bank ID)

TP53: https://www.rcsb.org/structure/8A9 (PDB ID: 8A92 Chain A). TLR4: https://www.rcsb.org/structure/ 2Z62 (PDB ID: 2Z62 Chain A). TNF: https://www.rcsb.org/structure/5UUI (PDB ID: 5UUI Chain A). ALB: https://www.rcsb.org/structure/6YG9 (PDB ID: 6YG9 Chain A). IL1B: https://www.rcsb.org/structure/5R8Q (PDB ID: 5R8Q Chain A). NFKB1: https://www.rcsb.org/structure/1SVC (PDB ID: 1SVC Chain P).

#### Protein preprocessing

The crystal structures of the six proteins were obtained by the *RCSB PDB* database (https://www.rcsb.org/). The protein preparation wizard module in Schrodinger software was used to process the obtained protein crystals (protein preprocess, regenerate states of native ligand, H-bond assignment optimization, protein energy minimization, and removal waters).

#### Ligand preprocessing

The 2D sdf structure file of Ellagic acid was processed by the LigPrep module in Schrodinger and all its 3D chiral conformations were generated.

#### Active site recognition

The SiteMap module in Schrodinger was used to predict the best binding site. Then, in the Receptor Grid Generation module of Schrodinger, the most appropriate Enclosing box was set to perfectly wrap the predicted binding sites, and the active sites of six proteins were obtained.

#### Molecular docking

Schrodinger Maestro 13.5 (February 2023 version) was used to perform molecular docking of the treated ellagic acid with the active sites of six proteins respectively (XP docking with the highest precision). The lower the score, the lower the binding free energy of ellagic acid and proteins, and the higher the binding stability.

#### Molecular mechanics generalized Born surface area (MM-GBSA) analysis

According to the MM-GBSA analysis of ellagic acid and the active sites of six proteins, MM-GBSA dG Bind can approximately represent the binding free energy between small molecules and proteins. The lower the binding free energy, the higher the binding stability of the ligand compound to the protein.

### Molecular dynamics simulation

To further optimize the binding mode of compound–protein complexes, we performed conventional molecular dynamics simulations by using the Desmond program. The OPLS4 force field was employed to parameterize the protein and small molecules, while the SPCE model was used for the water solvent. The protein-small molecule complex was placed in a cubic water box and solvated. The system's charge was neutralized by adding 0.150 M chloride and sodium ions. The energy of the system was initially minimized using the steepest descent minimization method for 50,000 steps. Subsequently, the positions of heavy atoms were restrained for NVT and NPT equilibration for an additional 50,000 steps. The system temperature was maintained at 300 K, and the system pressure was maintained at 1 bar. After completing the two equilibration stages, an unrestricted simulation was performed for 100 ns. The interactions were analyzed, and dynamic trajectory animations were generated using Maestro 2023.

### Saturation mutagenesis

#### Crystal selection

After flexible XP docking of EA with TP53, TLR4, and TNF-α proteins, MM-GBSA-optimized complex crystals were used as the research object for this saturation mutagenesis.

#### Selection of saturation mutagenesis sites

Based on the previous work, 6 amino acids interacting with EA on TP53 protein were selected as Saturation mutagenesis sites (LEU145, THR150, GLY154, THR155, CYS220, ASP228), 6 amino acids interacting with EA on TLR4 protein were selected as the Saturation mutagenesis sites (LEU117, THR136, ASN137, ALA139, ILE114, PHE144), 7 amino acids that interact with EA on TNF protein were selected as Saturation mutagenesis sites (ALA18, ARG32, PHE144, GLU146, GLY148, GLN149, VAL150). The Residue Scanning module in Schrodinger was adopted to set the amino acids at each site as all possible mutations (20 in total), and then the optimization model was set as Side-chain prediction with backbone minimization. All possible mutations were then performed on all sites.

#### Affinity calculation

The affinity of TP53, TLR4, and TNF-α proteins to Ellagic acid was evaluated by the affinity parameter. The TP53, TLR4, and TNF-α proteins in the complex were set as system A, and the Ellagic acid ligand was set as system B. The parameters of system A and system B were calculated. Ran the Python command: $SCHRODINGER/run residue_scanning_backend.py.

#### Saturation mutation results were plotted

Residue scanning viewer was used to combine and draw the results to show the line graph of ΔAffinity from low to high.

### Surface plasmon resonance analysis

Biacore T200 (GE Healthcare, USA) was used for real-time binding interaction studies. In this experiment, the method of CM5 chip amino coupling was used. TLR4 was first fixed to the Fc2 channel, and the Fc1 channel was used as a reference channel. The conditions of TLR4 coupling were as follows: the concentration of TLR4 was about 20 μg/mL, the system was pH4.0 sodium acetate solution, the chip was activated for 420 s, the 420 s was blocked with ethanolamine, and HEPES was used as the mobile phase of the coupled protein. Ellagic acid was diluted into 62.5, 31.25, 15.63, 7.813, and 3.909 μmol/L solutions with HEPES solution containing 5% DMSO (pH 7.4). Resatorvid was diluted into solutions of 125, 62.5, 31.25, 15.6 and 7.813 μmol/L. Resatorvid was determined under the following conditions: injection for 60 s, dissociation for 60 s, a flow rate of 30 μL/min, and no regeneration. Ellagic acid was determined under the following conditions: injection for 60 s, dissociation for 80 s, flow rate of 30 μL/min, and no regeneration.

## Conclusion

TP53, TLR4, and TNF-α are the potential targets of ellagic acid to exert an anti-RV effect, which may ultimately exert its anti-RV effect by regulating TLR4/NF-κB related pathway.

### Supplementary Information

Supplementary Information 1.

Supplementary Information 2.

Supplementary Information 3.

Supplementary Information 4.

## Data Availability

The datasets used and analyzed during the current study are available from the corresponding author upon reasonable request. Also, the datasets generated during the current study and Supplementary Information are available in the [Figshare] repository, [DOI:10.6084/m9.figshare.25060691].

## References

[1] Matthijnssens J (2022). ICTV virus taxonomy profile: Sedoreoviridae, 2022. J. Gen. Virol..

[2] Crawford SE (2017). Rotavirus infection. Nat. Rev. Dis. Primers.

[3] Lefkowitz EJ (2018). Virus taxonomy: The database of the international committee on taxonomy of viruses (ICTV). Nucleic Acids Res..

[4] Bartlett AV, Bednarz-Prashad AJ, DuPont HL, Pickering LK (1987). Rotavirus gastroenteritis. Annu. Rev. Med..

[5] Giri S (2019). Rotavirus gastroenteritis in Indian children < 5 years hospitalized for diarrhoea, 2012 to 2016. BMC Public Health.

[6] GBD 2016 Causes of Death Collaborators (2016). Global, regional, and national age-sex specific mortality for 264 causes of death, 1980–2016: A systematic analysis for the Global Burden of Disease Study 2016. Lancet..

[7] Weldegebriel G (2018). Impact of rotavirus vaccine on rotavirus diarrhoea in countries of east and southern Africa. Vaccine.

[8] Lestari FB, Vongpunsawad S, Wanlapakorn N, Poovorawan Y (2020). Rotavirus infection in children in Southeast Asia 2008–2018: Disease burden, genotype distribution, seasonality, and vaccination. J. Biomed. Sci..

[9] Cohen AL (2022). Aetiology and incidence of diarrhoea requiring hospitalisation in children under 5 years of age in 28 low-income and middle-income countries: Findings from the Global Pediatric Diarrhea Surveillance network. BMJ Glob. Health.

[10] GBD 2015 Mortality and Causes of Death Collaborators (2016). Mortality and Causes of Death Collaborators. Global, regional, and national life expectancy, all-cause mortality, and cause-specific mortality for 249 causes of death, 1980–2015: A systematic analysis for the global burden of disease study 2015. Lancet.

[11] Okafor CE, Ekwunife OI (2021). Introducing rotavirus vaccine in eight sub-Saharan African countries: A cost-benefit analysis. Lancet Glob. Health.

[12] Gower CM (2020). Vaccine-derived rotavirus strains in infants in England. Arch. Dis. Child..

[13] Xiang Q (2022). The bioactivity and applications of pomegranate peel extract: A review. J. Food Biochem..

[14] Caballero V (2022). Biodegradation of punicalagin into ellagic acid by selected probiotic bacteria: A study of the underlying mechanisms by MS-based proteomics. J. Agric. Food Chem..

[15] Yang Y (2023). Ellagic acid from pomegranate peel: Consecutive countercurrent chromatographic separation and antioxidant effect. Biomed. Chromatogr..

[16] BenSaad LA, Kim KH, Quah CC, Kim WR, Shahimi M (2017). Anti-inflammatory potential of ellagic acid, gallic acid and punicalagin A&B isolated from punica granatum. BMC Complement Altern. Med..

[17] Cota D, Patil D (2023). Antibacterial potential of ellagic acid and gallic acid against IBD bacterial isolates and cytotoxicity against colorectal cancer. Nat. Prod. Res..

[18] Acquadro S (2020). Punica granatum leaf ethanolic extract and ellagic acid as inhibitors of zika virus infection. Planta Med..

[19] Jaman MS, Sayeed MA (2018). Ellagic acid, sulforaphane, and ursolic acid in the prevention and therapy of breast cancer: Current evidence and future perspectives. Breast Cancer.

[20] Anitha P, Priyadarsini RV, Kavitha K, Thiyagarajan P, Nagini S (2013). Ellagic acid coordinately attenuates Wnt/β-catenin and NF-κB signaling pathways to induce intrinsic apoptosis in an animal model of oral oncogenesis. Eur. J. Nutr..

[21] Aggarwal BB, Shishodia S (2004). Suppression of the nuclear factor-kappaB activation pathway by spice-derived phytochemicals: Reasoning for seasoning. Ann. N. Y. Acad. Sci..

[22] Edderkaoui M (2008). Ellagic acid induces apoptosis through inhibition of nuclear factor kappa B in pancreatic cancer cells. World J. Gastroenterol..

[23] Chen J, Yang H, Sheng Z (2020). Ellagic acid activated PPAR signaling pathway to protect ileums against castor oil-induced diarrhea in mice: Application of transcriptome analysis in drug screening. Front. Pharmacol..

[24] Zhang R, Zhu X, Bai H, Ning K (2019). Network pharmacology databases for traditional Chinese medicine: Review and assessment. Front. Pharmacol..

[25] Pinzi L, Rastelli G (2019). Molecular docking: Shifting paradigms in drug discovery. Int. J. Mol. Sci..

[26] Collier TA, Piggot TJ, Allison JR (2020). Molecular dynamics simulation of proteins. Methods Mol. Biol..

[27] Lokhande KB (2023). Computational docking investigation of phytocompounds from bergamot essential oil against serratia marcescens protease and FabI: Alternative pharmacological strategy. Comput. Biol. Chem..

[28] Luan J (2022). Selectivity mechanism of BCL-XL/2 inhibition through in silico investigation. Phys. Chem. Chem. Phys..

[29] Gudkov AV, Komarova EA (2016). P53 and the carcinogenicity of chronic inflammation. Cold Spring Harb. Perspect Med..

[30] Tergaonkar V, Pando M, Vafa O, Wahl G, Verma I (2002). p53 stabilization is decreased upon NFkappaB activation: A role for NFkappaB in acquisition of resistance to chemotherapy. Cancer Cell.

[31] Xia Y (2009). Phosphorylation of p53 by IkappaB kinase 2 promotes its degradation by beta-TrCP. Proc. Natl. Acad. Sci. USA.

[32] Bhosle SM (2010). Augmentation of radiation-induced apoptosis by ellagic acid. Cancer Invest..

[33] Murphy SH (2011). Tumor suppressor protein (p)53, is a regulator of NF-kappaB repression by the glucocorticoid receptor. Proc. Natl. Acad. Sci. USA.

[34] Huyghe J, Priem D, Bertrand MJM (2023). Cell death checkpoints in the TNF pathway. Trends Immunol..

[35] Zhong C (2023). Phosphorylation by IKKβ promotes the degradation of HMGCL via NEDD4 in lung cancer. Int. J. Biol. Sci..

[36] Liu P (2022). Changes in humoral immunity, myocardial damage, trace elements, and inflammatory factor levels in children with rotavirus enteritis. Am. J. Transl. Res..

[37] Hakim MS (2018). TNF-α exerts potent anti-rotavirus effects via the activation of classical NF-κB pathway. Virus Res..

[38] Yusuf S (2019). The effect of zinc supplementation on pro-inflammatory cytokines (TNF-α, IL-1 and IL-6) in mice with *Escherichia coli* LPS-induced diarrhea. Iran. J. Microbiol..

[39] Lim KH, Staudt LM (2013). Toll-like receptor signaling. Cold Spring Harb Perspect. Biol..

[40] Xiong T (2022). Ganluyin ameliorates DSS-induced ulcerative colitis by inhibiting the enteric-origin LPS/TLR4/NF-κB pathway. J. Ethnopharmacol..

[41] Vaure C, Liu Y (2014). A comparative review of toll-like receptor 4 expression and functionality in different animal species. Front. Immunol..

[42] Wang X (2021). Shenling baizhu powder inhibits RV-SA11-induced inflammation and rotavirus enteritis via TLR4/MyD88/NF-κB signaling pathway. Front. Pharmacol..

[43] Chen X (2020). Ziyuglycoside II inhibits rotavirus induced diarrhea possibly via TLR4/NF-κB pathways. Biol. Pharm. Bull..

[44] Wardill HR (2016). TLR4-dependent claudin-1 internalization and secretagogue-mediated chloride secretion regulate irinotecan-induced diarrhea. Mol. Cancer Ther..

[45] Folkard DL (2014). Suppression of LPS-induced transcription and cytokine secretion by the dietary isothiocyanate sulforaphane. Mol. Nutr. Food Res..

[46] Ge Y (2013). Rotavirus NSP4 triggers secretion of proinflammatory cytokines from macrophages via toll-like receptor 2. J. Virol..

