# Supplementary material for: Network pharmacology, computational biology integrated surface plasmon resonance technology reveals the mechanism of ellagic acid against rotavirus
Source: Sci Rep. 2024 Mar 30;14:7548. doi: 10.1038/s41598-024-58301-6 (PMC10981743; doi:10.1038/s41598-024-58301-6)
Supplement: Supplementary file 1 — Supplementary Information 1. [file 41598_2024_58301_MOESM1_ESM.docx]

**Conformational evolution of ellagic acid rotatable bond (RB)**

The conformational evolution of each RB in ellagic acid within the entire simulated locus (0-100 ns) is shown in Figure S1. The dial diagram describes the twisting pattern throughout the simulation. The starting point of the simulation is in the center of the radial graph, and the evolution of time is plotted radially outward. The bar chart summarizes the data on the dial chart by showing the probability density of torsion. EA with TP53 and TLR4 showed similar torsional conformation in red, yellow, and green RB, while the TLR4 protein activity pocket is smaller so that the blue RB from -45°-45° cannot be twisted. The twist conformation of EA and TNF was completely different from the first two.


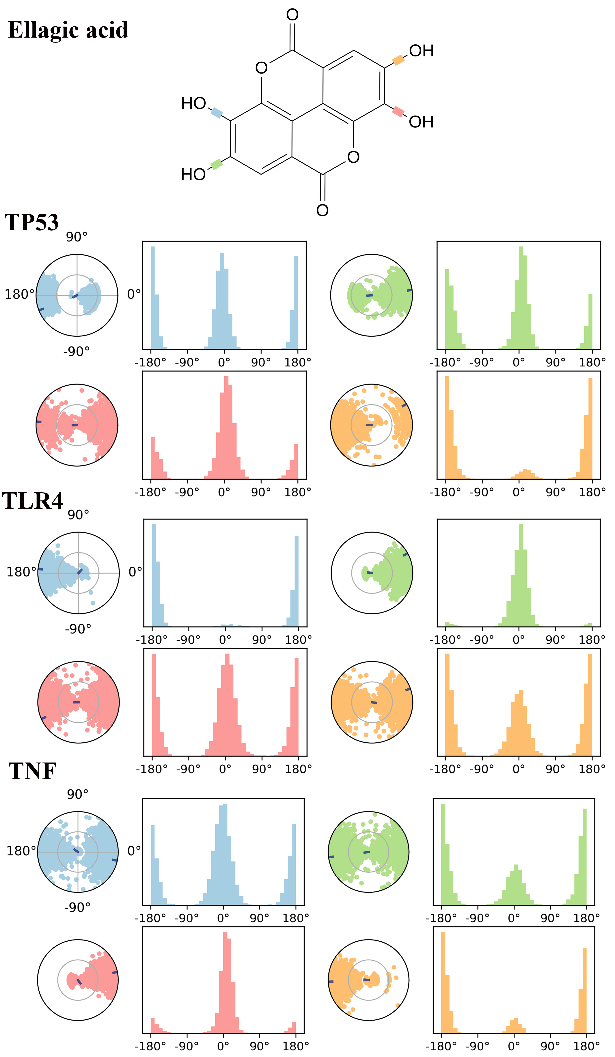


Figure S1. Torsion conformation for each rotatable bond of ellagic acid.

**Two-dimensional structure of Δaffinity at top3 sites of TP53, TLR4 and TNF-α**

It is difficult to find significant changes in the two-dimensional structure of the top3 sites with Δaffinity of TP53, TLR4, and TNF-α, which indicates that the mutation mainly affects non-covalent bonds such as hydrogen bonds and salt bridges (Supplementary Fig.S2).


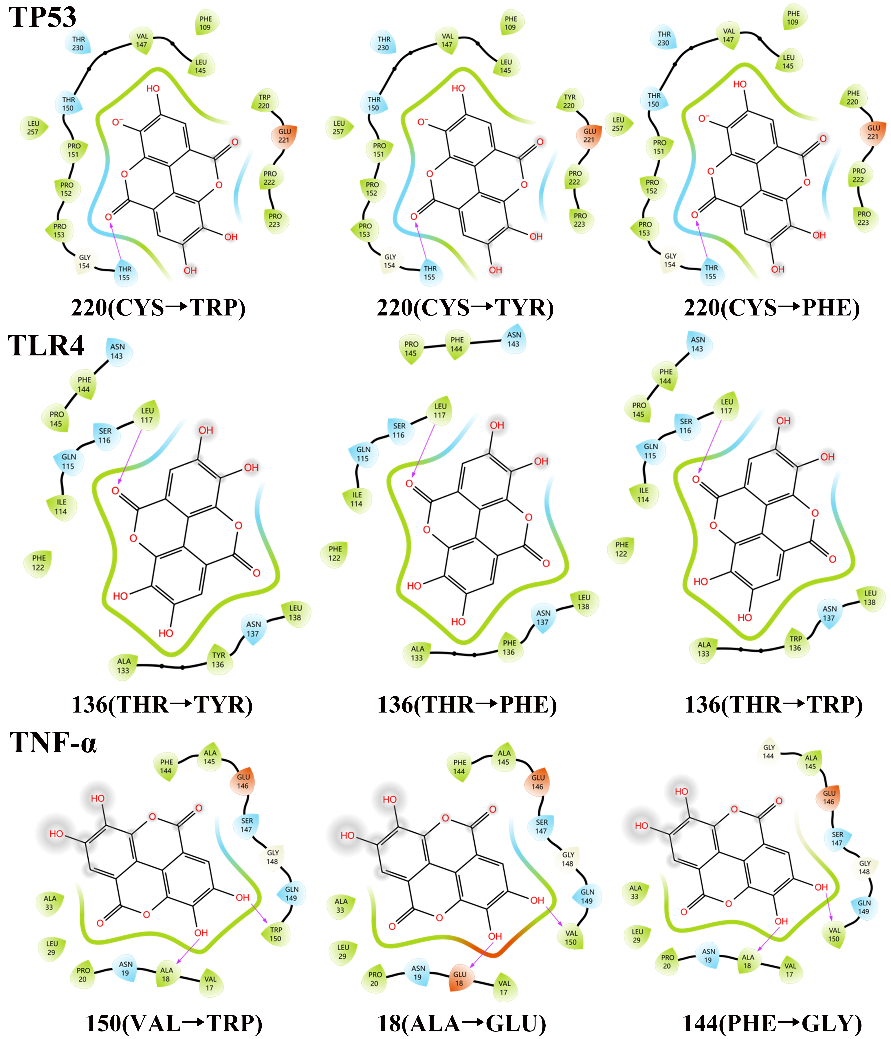


Figure S2. Interaction of TOP3 saturation mutagenesis affinity at key binding sites.
